# Effect of cell microenvironment on the drug sensitivity of hepatocellular cancer cells

**DOI:** 10.18632/oncotarget.27910

**Published:** 2021-03-30

**Authors:** Bhaskar Bhattacharya, Daniel Q. Huang, Sarah Hong Hui Low, Gim Hwa Tan, Min Ji Han, Sanamerjit Singh, Benny Tang, Sheng Chun Chang, Joey Sze Yun Lim, Mohd Feroz Mohd Omar, Yock Young Dan, Richie Soong

**Affiliations:** ^1^Cancer Science Institute of Singapore, National University of Singapore, Singapore; ^2^Pascific Laboratories, Singapore; ^3^Department of Medicine, National University of Singapore, Singapore; ^4^Division of Gastroenterology and Hepatology, National University Health System, Singapore; ^5^Integrated Science, University of British Columbia, Vancouver, Canada

**Keywords:** drug development, microenvironment, glycolysis

## Abstract

The native hepatocellular cancer (HCC) microenvironment is characterized by more hypoxic, hypoglycemic, and acidic conditions than those used in standard cell culture. This study aimed to investigate whether HCC cells cultured in more native conditions have an altered phenotype and drug sensitivity compared to those cultured in standard conditions. Six HCC cell lines were cultured in “standard” (21% O_2_, 25 mM glucose) or more “native” (1% O_2_, 5 mM glucose, 10 mM lactate) conditions. Cells were assessed for growth rates, cell cycle distribution, relevant metabolite and protein levels, genome-wide gene expression, mitochondrial DNA sequence and sensitivity to relevant drugs. Many differences in cellular and molecular phenotypes and drug sensitivity were observed between the cells. HCC cells cultured in native conditions had slower doubling times, increased HK2 and GLUT, lower PHDA and ATP levels, and mutations in mitochondrial DNA. Thirty-one genes, including the hypoxia-associated *NDRG1*, were differentially expressed between the cells. HCC patients in The Cancer Genome Atlas (TCGA) with tumors with a high score based on these 31 genes had a poorer prognosis than those with a low score (*p* = 0.002). From 90 comparisons of drug sensitivity, increased resistance and sensitivity for cells cultured in native conditions was observed in 14 (16%) and 8 (9%) comparisons respectively. In conclusion, cells cultured in more native conditions can have a more glycolytic and aggressive phenotype and varied drug sensitivity to those cultured in standard conditions, and may provide new insights to understanding tumor biology and drug development.

## INTRODUCTION

Hepatocellular carcinoma (HCC) is the third leading cause of cancer related death worldwide, with a poor median survival time after diagnosis of six months [1]. The benefits of surgical treatment of HCC is often limited, as up to 54% patients exhibit recurrent disease [2]. The current first line systemic therapy for advanced HCC is sorafenib or lenvatinib, which gives an estimated survival benefit of around three months [3]. For patients who have progressed on sorafenib, regorafenib prolongs survival by an additional two to three months [4]. Hence, there is an unmet need for more efficacious systemic treatments, however the majority of Phase III clinical trials in HCC have notoriously failed [5].

The glucose supply of most solid tumors is lower than in physiological tissue, due to a disorganized vascular supply with tortuous, irregular vessels that have irregular blood flow [6]. HCC have been reported to have low glucose, glycerol 3- and 2-phosphate levels which is consistent with metabolic remodeling and increased glycolysis [7]. The hypoglycemic environment contributes to metabolic stress, promotes autophagy, and activates stress signaling pathways [8].

The HCC microenvironment is also characterized by significant hypoxia, with a median tumor pO2 of only 6 mmHg compared to 30 mmHg in normal liver [9]. The reduced oxygen concentration has been attributed to abnormalities in the tumor microvasculature, as well as increased diffusion distances [10]. Hypoxia is a key mechanism that stimulates angiogenesis in HCC through upregulation of vascular endothelial growth factor (VEGF) gene transcription and improved mRNA stability [11]. Hypoxia inducible factors (HIFs) also promote glycolysis, and are more prominent in aggressive HCC subtypes [12].

Lactate is also high in the HCC microenvironment, owing to the Warburg effect under which tumor cells convert glucose into lactate with an overall less production of ATP compared to mitochondrial oxidative phosphorylation [13]. Cells with elevated lactate are more frequently arrested at the G0/G1 cell cycle phase, where metabolic requirements are lower, and cells are more primed for autophagy [14]. The acidic tumor environment also promotes cell proliferation and invasion [14]. Lactate also stabilizes NDRG3 which binds c-Raf, leading to activation of the Raf-ERK pathway and angiogenesis [15].

Conditions used in standard cell culture today were historically established from studies more concerned with achieving stable cell immortality and growth [16]. As understanding of the tumor microenvironment has increased over time, it has become apparent that the historical culture conditions do not reflect the native tumor microenvironment well, including having increased glucose, increased oxygen, and reduced lactic acid concentrations. Indeed, we previously showed that gastric cancer cells cultured in low glucose (5 mM) compared to standard high glucose (25 mM) levels had an increased resistance to 5-fluorouracil and carboplatin - concomitant with increased glycolysis and mitochondrial mutation [17]. This led us to postulate that culturing cells in glucose, oxygen, and lactate conditions that are more consistent with the native tumor microenvironment may provide a better assessment of drug sensitivity, and help lead to higher success rates in drug development. The goal of this study was to characterize the effect of culturing HCC cells in native compared to standard culture conditions on cellular and molecular phenotypes and drug sensitivity.

## RESULTS

### Cell growth and cell cycle distribution

**Figure 1:**
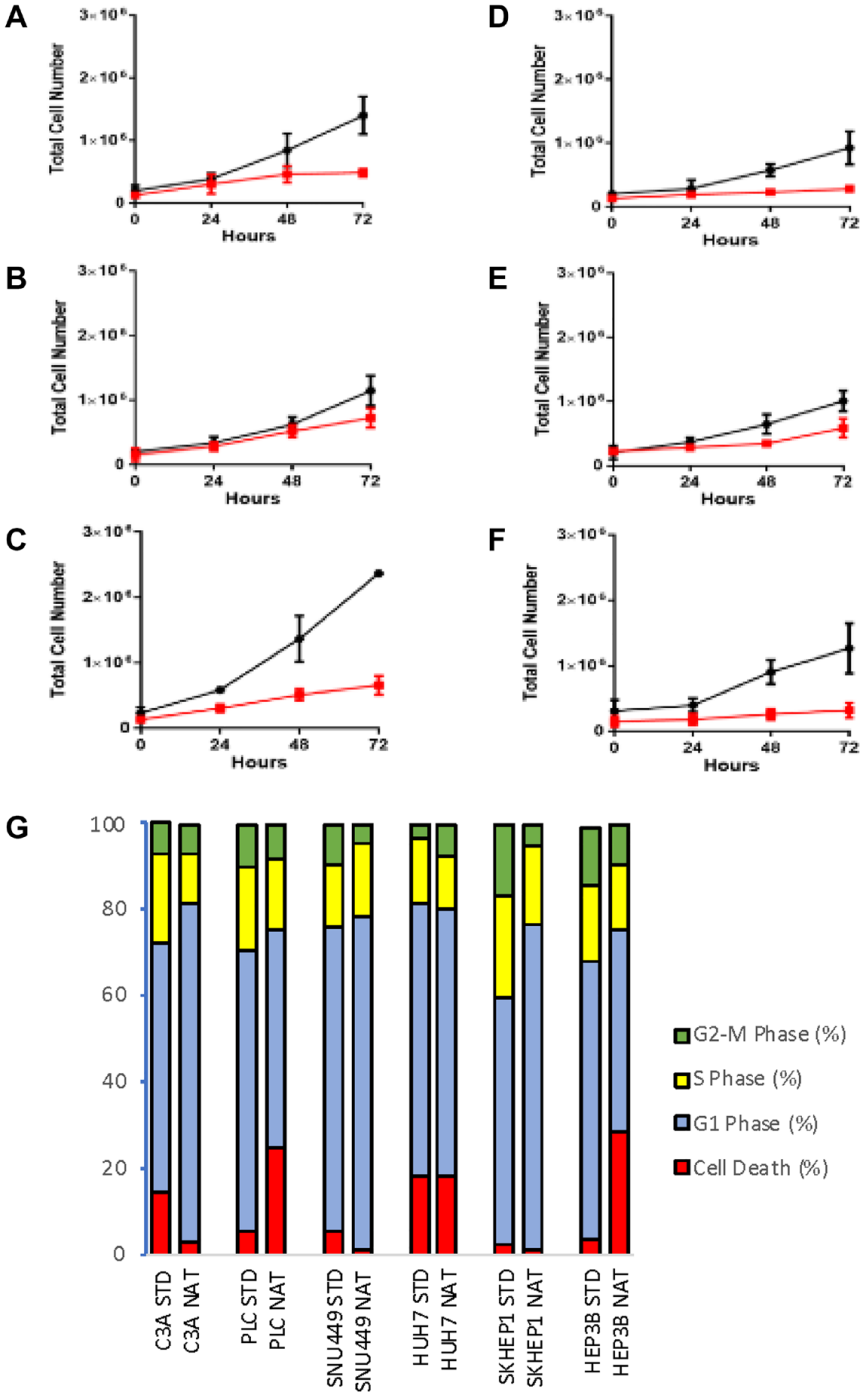
Cell phenotypes of cells cultured in standard and native conditions. Cell proliferation evaluated by MTS assay of cells grown in standard (black line) and native (red line) conditions in (**A**) C3A (**B**) PLC, (**C**) SNU449, (**D**) SKHEP1, (**E**) HUH7, and (**F**) HEP3B cells. (**G**) Cell cycle profile of respective cells grown in standard (STD) and native (NAT) conditions. Data shown as mean ± SD from three independent experiments.

HCC cells cultured in native compared to standard conditions grew at a significantly slower rate for all cells tested (Figure 1A–1F). PLC and HEP3B cell lines had an increased sub G1 phase (Figure 1G), suggestive of increased DNA degradation and cell death, as well as increased G2-M and reduced G1 phase distribution. C3A, SNU449, SKHEP1 cells had an increased G1 fraction, indicative of increased G1 arrest.

### ATP, ROS, and lactate production

**Figure 2:**
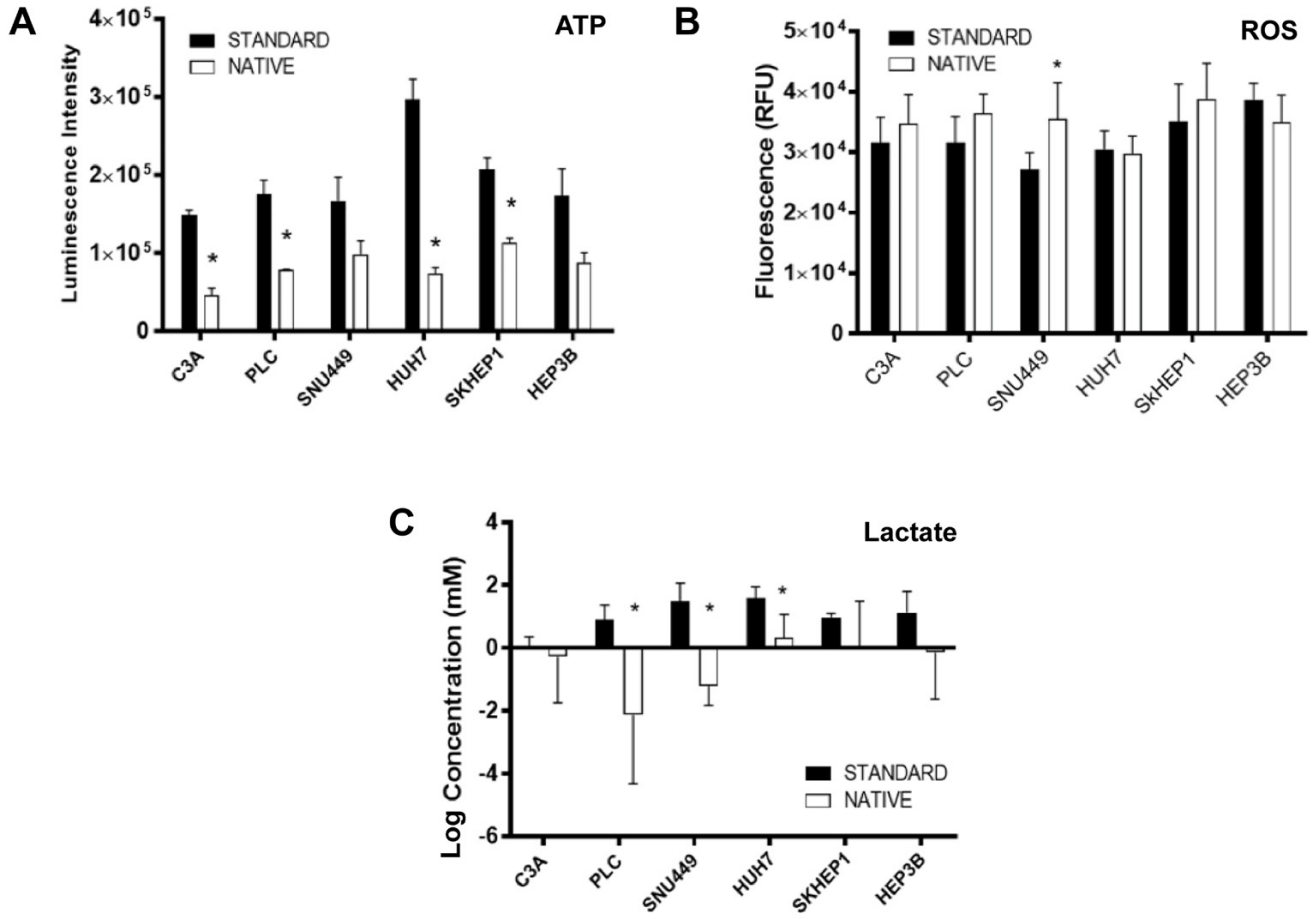
Molecular phenotypes of cells cultured in standard and native conditions. (**A**) ATP (**B**) ROS, and (**C**) lactate production relative to baseline levels, in respective cell lines cultured in native (white bars) and standard (black bars) conditions. Data shown as mean ± SD from three independent experiments.

For cells cultured in native compared to standard conditions, ATP production was consistently reduced, with the reduction being statistically significant in C3A, PLC, HUH7 and SKHEP1 cells (Figure 2A). ROS production was significantly higher in SNU449 cells cultured in native compared to standard conditions, while there was no significant difference for the other cell lines (Figure 2B). Normalized to baseline levels, lactic acid production was lower in all cells cultured in native conditions compared to standard conditions, with the differences being significant in PLC, SNU449 and HUH7 cells (Figure 2C).

### Glycolysis, glucose transporters, PI3K, stress, and cell death proteins

**Figure 3:**
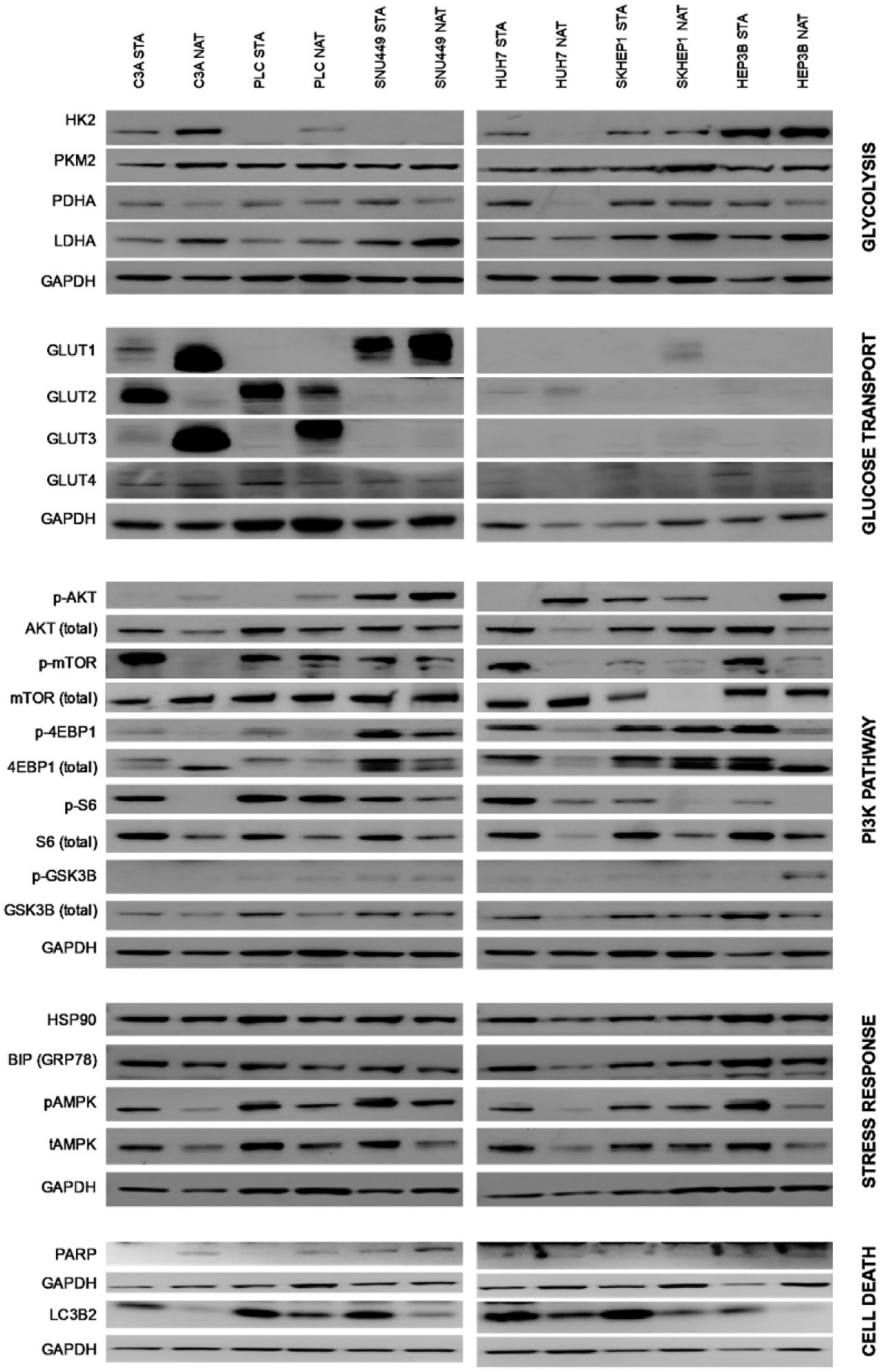
Protein expression of cells cultured in standard and native conditions. The proteins are grouped according to their roles in glycolysis, glucose transport, PI3K pathway, stress response, and cell death. Also indicated are respective cell lines cultured in native (NAT) and standard (STA) conditions. Representative immunoblots shown of three independent experiments.

There were many differences between cells cultured in native and standard conditions in the levels of proteins involved in glycolysis, glucose transport, PI3K pathway signaling, stress response and cell death (Figure 3). When cultured in native conditions, HK2 was higher in C3A and PLC cells and lower in HUH7 cells (Figure 3). Cells generally had lower PDHA and higher LDHA levels, with the exception of PLC and SKHEP1 for PDHA, and HUH7 for LDHA. GLUT1 was elevated in C3A, SNU449 and SKHEP1 cells, and GLUT3 in C3A and PLC cells. GLUT2 was reduced in C3A and PLC cells. AKT phosphorylation was generally increased in all cells except for SKHEP1, with total AKT levels lower in C3A, HUH7 and HEP3B cells. mTOR phosphorylation levels were generally lower in all cell lines, while total mTOR levels were unchanged, with the exception of lower total mTOR in SKHEP1 cells cultured in native conditions. 4EBP phosphorylation was lower in C3A, PLC and HEP3B cells, while total 4EBP levels were higher in C3A and lower in HUH7, SNU449 and HEP3B cells. S6 phosphorylation was lower in C3A, SKHEP1, and HEP3B, and HUH7 cells. In HUH7, total S6 levels were also lower. For GSK3B, phosphorylation was higher in HEP3B cells, while total protein levels were lower in PLC, HUH7, and HEP3B cells. There were few differences in stress response proteins, with the exception of lower GRP78 in HUH7and PLC cells. Intriguingly, C3A and HEP3B cells had lower total AMPK and LC3B2, suggestive of increased autophagy or a higher turnover of autophagosomes [18]. Total AMPK levels were also lower in HUH7 and LC3B2 levels lower in SNU449 cells. As observed by measurement of PARP, there was no evidence of differences in baseline apoptosis.

### Gene expression

**Figure 4:**
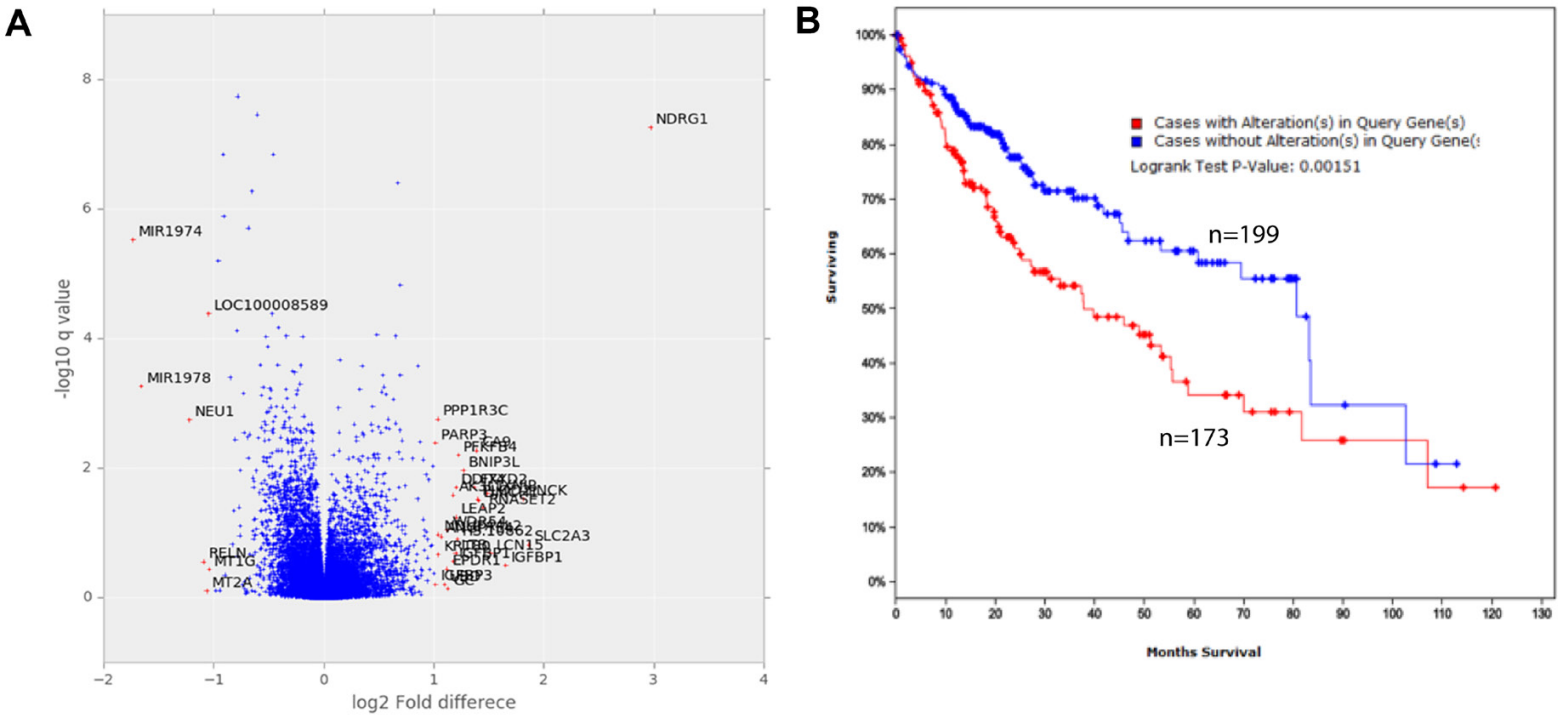
Gene expression of cells cultured in standard and native conditions. (**A**) Fold-change and *q*-values of the difference in levels of all genes. Analysis was performed from triplicate samples representing each condition. (**B**) Survival of HCC patients that have dysregulation in the top 31 dysregulated genes (red curve, *n* = 172) compared to those without (blue curve, *n* = 199) graphed according to the Kaplan–Meier method. The difference between groups is significant (*p* = 0.00151).

From gene array analysis of more than 31,000 genes, thirty-one genes were found to be differentially expressed in native conditions compared to standard conditions (Figure 4A, Table 1). Most notable amongst these was N-myc downstream-regulated gene-1 (*NDRG1*) which was elevated 7.8-fold (*p* < 0.001) in cells cultured in native conditions. Interestingly, interrogation of the expression levels of HCC patients in “The Cancer Genome Atlas” (TCGA) dataset revealed 173 patients with dysregulation in the 31 genes had median survival time of 37.75 months compared to 80.68 months for 199 patients without dysregulation (*p* = 0.002) (Figure 4B). Dysregulation in these 31 genes did confer a survival difference when evaluated in other TCGA datasets for other cancers, such as stomach, lung, colon, breast, endometrial and cervical cancer.

**Table 1:** Genes significantly differentially expressed in cells cultured in native compared to standard conditions, ranked according to p-value

No	Gene Symbol	Gene Description	*p*	Difference
1	NDRG1	N-myc downstream regulated 1	5.51E-08	7.836
2	PPP1R3C	protein phosphatase 1, regulatory subunit 3C	1.75E-03	2.048
3	NEU1	sialidase 1 (lysosomal sialidase)	1.80E-03	0.429
4	PARP3	poly (ADP-ribose) polymerase family, member 3	4.11E-03	2.018
5	CA9	carbonic anhydrase IX	5.35E-03	2.611
6	PFKFB4	6-phosphofructo-2-kinase/fructose-2,6-biphosphatase 4	6.28E-03	2.333
7	BNIP3L	BCL2/adenovirus E1B 19kDa interacting protein 3-like	1.09E-02	2.408
8	DDIT4	DNA-damage-inducible transcript 4	1.97E-02	2.298
9	FXYD2	FXYD domain containing ion transport regulator 2	1.98E-02	2.581
10	TXNIP	thioredoxin interacting protein	2.49E-02	2.806
11	AK3L1	adenylate kinase 4	2.60E-02	2.261
12	PNCK	pregnancy up-regulated non-ubiquitously expressed CaM kinase	2.99E-02	3.505
13	PLOD2	procollagen-lysine, 2-oxoglutarate 5-dioxygenase 2	3.02E-02	2.631
14	LIMCH1	LIM and calponin homology domains 1	3.17E-02	2.650
15	RNASET2	ribonuclease T2	4.05E-02	2.732
16	LEAP2	liver expressed antimicrobial peptide 2	5.73E-02	2.297
17	WDR54	WD repeat domain 54	9.04E-02	2.174
18	NDUFA4L2	NADH dehydrogenase (ubiquinone) 1 alpha subcomplex, 4-like 2	1.06E-01	2.055
19	ANGPTL4	angiopoietin-like 4	1.14E-01	2.092
20	SLC2A3	solute carrier family 2 (facilitated glucose transporter), member 3	1.52E-01	3.640
21	LCN15	lipocalin 15	2.04E-01	2.860
22	LTB	lymphotoxin beta (TNF superfamily, member 3)	2.04E-01	2.298
23	KRT80	keratin 80	2.16E-01	2.054
24	IGFBP1	insulin-like growth factor binding protein 1	2.74E-01	2.264
25	RELN	reelin	2.79E-01	0.468
26	EPDR1	ependymin related protein 1 (zebrafish)	3.60E-01	2.170
27	MT1G	metallothionein 1G	3.62E-01	0.485
28	IGFBP3	insulin-like growth factor binding protein 3	6.20E-01	2.018
29	UBD	ubiquitin D	6.25E-01	2.137
30	GC	group-specific component (vitamin D binding protein)	7.16E-01	2.186
31	MT2A	metallothionein 2A	7.89E-01	0.478

### Mitochondrial DNA mutations observed in NAT HCC cell lines

Mutations were noted in the cell lines cultured in native conditions, tested after subculturing and stabilization for 3 months (Table 2). These include mutations in mitochondrially encoded 16S RNA (MT-RNR2), mitochondrially encoded NADH: Ubiquinone Oxidoreductase Core Subunit 1 and 6, (MT-ND1) and (MT-ND6) respectively. NADH dehydrogenase subunit (ND) represents a core subunit of the mitochondrial membrane respiratory chain NADH dehydrogenase (Complex I) and performs a key role in energy metabolism, apoptosis and proliferation [19]. Mutations in ND1 have been previously described in HCC [20].

**Table 2:** Mitochondrial mutations detected in the HCC cells cultured under native (NAT) and standard (STA) conditions

Symbol	Gene	Variant	Type	Cell Line
MT-RNR2	mitochondrial ribosomal RNA	302 A>AC, ACC	insertion	SKHEP1 NAT
MT-RNR2	mitochondrial ribosomal RNA	302 A>AC, ACC	insertion	SNU449 STA
MT-RNR2	mitochondrial ribosomal RNA	302 A>AC, ACC	insertion	SNU449 NAT
MT-RNR2	mitochondrial ribosomal RNA	2487 A>C	SNV	PLC NAT
MT-ND1	Mitochondrially Encoded NADH:Ubiquinone Oxidoreductase Core	2487 A>C	SNV	HUH7 NAT
MT-ND6	Mitochondrially Encoded NADH:Ubiquinone Oxidoreductase Core Subunit 6	16188 CT>C	deletion	HUH7 NAT

### Drug sensitivity

**Figure 5:**
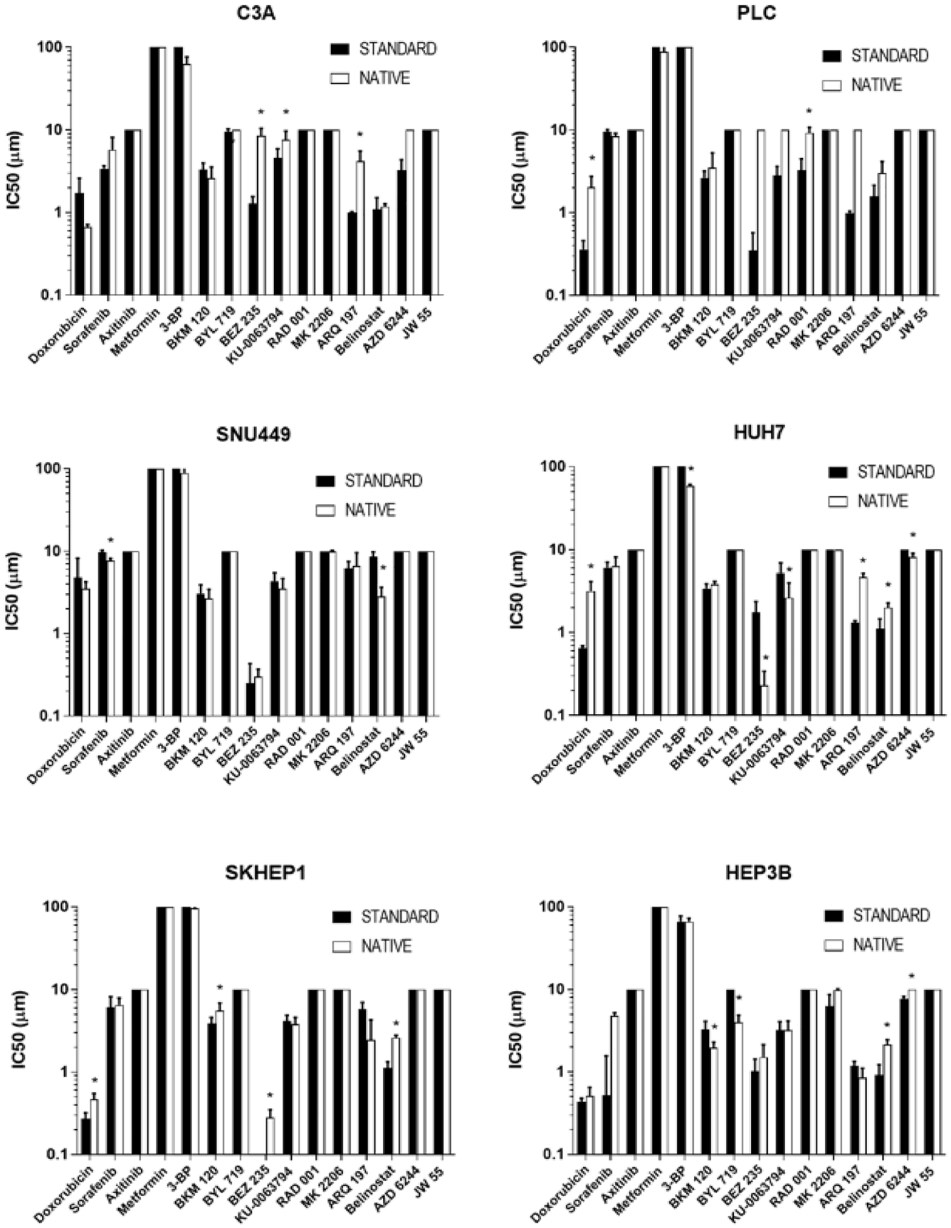
Drug sensitivity of cells cultured in standard and native conditions. The IC_50_ values of respective drugs (x-axis) of respective cells (respective charts) cultured in native (white bars) and standard (black bars) conditions. An asterisk (^*^) indicates drugs for which there are significant differences (*p* < 0.05) in IC_50_ values between native and standard conditions. Data shown are mean log IC_50_ ± SD from eight independent experiments.

From 90 comparisons of drug sensitivity, comprising 15 drugs in 6 cell lines, 22 (21%) significant differences were observed between cells cultured in native and standard conditions (Figure 5). These included increased resistance (higher IC_50_ concentrations) in 14 comparisons, namely for BEZ235, KU-0063794, and ARQ197 in C3A cells, doxorubicin and RAD001 in PLC cells, doxorubicin, ARQ197, and belinostat in HUH7 cells, doxorubicin, BKM120, BEZ235, and belinostat in SKHEP1 cells, and belinostat and AZD6244 in HEP3B cells. Cells were more sensitive (lower IC_50_ concentrations) in 8 comparisons, namely for sorafenib and belinostat in SNU499 cells, 3-BP, BEZ235, KU-0063794, and AZD6244 in HUH7 cells, and BKM120 and BYL719 in HEP3B cells.

## DISCUSSION

It has been long acknowledged that the tumor microenvironment influences a host of oncogenic responses such as immune evasion, disease progression, creation of a pre-metastatic niche, and drug resistance [21, 22]. This effect of the microenvironment may be attributed to a multitude of factors, such as tumor architecture and vasculature, presence of small molecules and cytokines, altered cellular signaling and gene expression due to an acidic and hypoxic milieu, and dysregulation of essential metabolites, for example glucose and lactate [21]. Indeed, many laboratories including ours have demonstrated that altering glucose or oxygen (hypoxia) concentrations is associated with changes in chemosensitivity of cancer cells [17, 23]. However, the collective outcome of some of the key components of tumor metabolism, such as glucose, lactate and hypoxia, on drug response in cancer cells have not been elucidated. In this study, we set out to explore the collective effect of the tumor microenvironment by creating a “native” cell-culture platform comprising physiological glucose levels, hypoxia and lactic acidosis, and examining their effect on cellular and molecular changes, and sensitivity to standard-of-care and targeted agents in HCC cells.

All HCC cells cultured in “native” conditions displayed slower doubling time compared to the cells cultured in standard conditions. HCC is a complex disease with multiple etiologies, the most notable being Hepatitis B or C, alcoholic liver disease, and non-alcoholic steatohepatitis [24]. While the clinical growth pattern of HCC has been reported to be variable [25], it has also been suggested that HCC does not grow in an exponential manner [26], especially early-stage tumors, owing to the nutrient-deprived microenvironment. As the native culture condition mimics some of the nutrient-deprived features of HCC microenvironment, the slower doubling time of the cell lines in such an environment may be expected.

The ability of tumor cells under nutrient and stress conditions to inhibit mTOR signaling and reducing protein synthesis to conserve energy is well documented [27]. Inhibition of mTOR is associated with decreased translation of proteins necessary for G1/S transition leading to an accumulation of cells in G1 phase [28]. Indeed, one of the more distinctive differences observed in this study was that many HCC cells cultured in native conditions exhibited lower levels of mTOR and 4EBP1 compared to standard conditions consistent with an observed increased G1 accumulation, and suggestive of an energy conservation mode. It has also been reported that glucose deprivation leads to an activation of AMPK as a result of an altered AMP:ATP ratio [29], which in turn inhibits mTOR signaling. However, in the present study, culturing in native conditions led to no difference or a decrease in levels of AMPK, despite reductions in levels of ATP (Figure 2A). One potential explanation is that whilst levels of glucose was reduced in native conditions compared to standard, glutamine and other growth factors were kept unchanged which perhaps prevented severe metabolic instability. Also, it has been reported that glucose deprivation can inhibit mTOR in AMPK null conditions in a Rag GTPase-dependent process [30]. This suggests an increase in AMPK may not always be necessary for mTOR inhibition in nutrient-deprived conditions.

The stress-adaptation of HCC cells in native conditions is also evidenced by unchanged levels of HSP90 and GRP78 compared to standard conditions. Cells cultured in the native environment generally had higher HK2, increased GLUT, lower PHDA (Figure 3), and lower ATP levels (Figure 2A), which is consistent with being cultured in lower glucose concentrations and having increased glycolytic activity. This observation is consistent with HCC being a glycolytic cancer [7]. Such changes observed would be expected to be associated with increase lactate accumulation. However, lower levels of lactate were detected in culture media of cells cultured in native conditions compared to standard conditions (Figure 2C). One explanation is that under reduced glucose environment, such as in native condition, and pre-existing high lactate concentration in culture media, the lactate produced as a by-product of glycolysis is re-used by the cells as a potential fuel to support survival. Lactate has been recognized as an important signaling molecule in the tumor microenvironment [31]. Furthermore, re-uptake of lactate in glycolytic conditions has been reported in human cancers [32, 33].

There were multiple differences in drug sensitivity between cells cultured in native and standard conditions, with a significantly increased resistance and sensitivity observed in 14 and 8 of 90 comparisons respectively (Figure 5). It can be argued that the increased resistance of cells cultured in native conditions to some drugs could be attributable to the slower growth rates of the cells, as slower cell growth rates are well known to result in increased drug resistance measurements [34]. However, the observation of increased sensitivity for other drugs in the same cells is indicative of an influence beyond growth rate alone. A clear pattern of sensitivity or resistance did not emerge which could be accounted for the limited number of compounds used in this study or the absence of combination regimens. However, an interesting observation was made with doxorubicin, a standard-of-care drug for HCC. Most of the HCC cell lines cultured in native conditions had higher IC_50_ values for doxorubicin compared to standard condition, and it is well documented that majority of HCC patients are intrinsically resistant to single agent doxorubicin treatment [35]. Numerous mechanisms of doxorubicin resistance in HCC have been reported [36], one of them being overexpression of *NDRG1* gene [37]. Additionally, overexpression of *NDRG1* has also been shown to be a poor prognostic marker in HCC, correlating with vascular invasion, recurrence, metastases, and poorly differentiated tumors [37]. *NDRG1* is downstream of the proto-oncogene N-Myc, which is upregulated by hypoxia [38], and also a downstream target of p53, which is required for p53-mediated caspase apoptosis [39]. Suppression of *NDRG1* by siRNA or pharmacological intervention sensitized HCC cells to doxorubicin both *in vitro* and *in vivo* [37, 40]. In our study, HCC cells cultured in native conditions significantly overexpressed *NDRG1* (Figure 4), which is consistent with HCC tumor biology and its associated resistance to doxorubicin. Therefore, drug testing performed in standard, non-hypoxic conditions may not provide reliable information due to an artificial suppression of oncoproteins, as observed with NDRG1 in the current study.

In addition to *NDRG1*, gene expression array analysis revealed 31 genes to be significantly differentially expressed between cells cultured in native compared to standard conditions (Figure 4A, Table 1). Interestingly, interrogation of HCC, stomach, lung, colon, breast, endometrial and cervical cancer datasets in the TCGA, revealed patients with higher levels of these 31 genes has a significantly shorter survival time than those with low levels. These results suggest the 31 genes could have a role in determining malignancy and/or drug resistance in these cancer types. However, it is important to recognize such a role requires further validation and delineation of mechanism of involvement, which awaits further study.

Given the central role of the mitochondria in regulating cellular bioenergetics and metabolism, it was decided to examine the integrity of the mitochondrial genome of HCC cells cultured in native conditions compared to standard culture conditions. Mutations in *MT-RNR2*, *MT-ND1*. and *MT-ND6* mitochondrial genes were observed in three out of six HCC cells cultured in native conditions (Table 1). Mitochondrial *MT-RNR2* encodes for the polypeptide, humanin, which possesses anti-apoptotic functions [41]. The relevance of this particular mutation in HCC cells cultured under native condition is not clearly understood. However, it can be speculated that this particular aberration provides a cytoprotective effect under stressful microenvironment conditions, as well as conferring a chemo-resistant phenotype. Indeed, the cytoprotective role of humanin have been reported in Alzheimer’s disease, with humanin suggested as a component of mitochondrial stress signaling [42].

In conclusion, we cultured HCC cells in its native environment compared to standard culture conditions, and observed phenotypic and molecular signatures of cells in native conditions pathologically similar to human HCC. The study highlights two important points, a) new insights in pathogenesis could be gained by culturing cells in conditions closer to physiological conditions, and b) a better idea of therapeutic response may be obtained by screening experimental agents in cancer cells grown in a microenvironment similar to actual disease setting. Culturing cells in their native conditions has the potential to identify therapy, either single agent or combination, which would have otherwise been considered ineffective under the currently used artificial environment. Additionally, novel targets may also be identified under native conditions in addition to refining our existing knowledge of tumor biology. Nonetheless, it is important to keep in perspective the exhaustive work of manually concurrently culturing cells in standard and native conditions, and conducting the diverse interrogations, limited this study to understanding of a few cell lines, mechanisms and drugs. Future studies incorporating high-throughput culturing and screening methods, and the testing of other hypotheses and timepoints will help to reveal the scope of generalizability of these findings.

## MATERIALS AND METHODS

### Cell lines

C3A, PLC, SKHEP1, HEP3B, SNU449 cells from American Type Culture Collection (Manassas, VA, USA), and HUH7 cells from the Japanese Collection of Research Bioresources (Tokyo, Japan) were obtained either directly, or through collaboration. The identity of the cells was confirmed using the GenePrint 10 System (Promega, Madison, WI), and the cells were regularly confirmed to lack mycoplasma using the LookOut Mycoplasma Elimination Kit (Sigma Aldrich, St. Louis, MO, USA). Standard culture conditions consisted of those recommended by respective suppliers, including media containing 25 mM glucose at pH7 (Thermo Fisher Scientific, Waltham, MA, USA), and incubation at standard oxygen levels (21%). For native culture conditions, media lacking glucose was obtained and supplemented with 5 mM glucose and 10 mM lactic acid (Thermo Fisher Scientific). Cells were incubated under 1% oxygen. All procedures for standard culture conditions were performed in a M371 incubator (Thermo Fisher Scientific) and native culture in a Ruskinn InvivO2 400 Hypoxia Workstation (Baker, Sanford, ME, USA). HCC cell lines were made to adapt by continuous sub-culturing in the native conditions in tandem with cells in standard conditions for a period of 3 months before commencing experimental procedures.

### Compounds

Doxorubicin, sorafenib, axitinib, metformin, 3-BP, BKM120, BYL719, BEZ235, KU-0063794, RAD001, MK2206, ARQ197, Belinostat, AZD6244, and JW55 were obtained from Selleck Chemicals (Houston, TX, USA). All compounds were diluted to stock solutions and stored according to supplier recommendations.

### Assessment of cell phenotypes

Assessment of cell proliferation, drug sensitivity, cell cycle distribution, gene expression arrays, and levels of selected proteins, ATP, ROS and lactate, was performed under standard protocols and as described previously [17, 43].

### Assessment of gene expression

RNA was extracted from cell lines with the RNeasy Mini Kit (Qiagen, Hilden, Germany) according to the manufacturer’s instructions. Microarray analysis was performed using the Infinium HumanOmniExpress-12 v3 Expression BeadChip kit (Illumina). The BeadChip was scanned on the HiScan system (Illumina). Gene expression analysis was performed in R v3.2.2. (http://www.r-project.org) within the R Bioconductor environment v3.1 [44]. Specifically, illuminaio v0.14.0 [45] was used for import of the Illumina BeadArray data, while QC, Variance stabilizing transform (VST) and Quantile normalization were performed using Lumi 2.24.0 [46]. Differential gene expression was performed using limma via Multiple Experiment Viewer (MeV) v4.9.0 [47]. Genes with a significant difference in expression were those having of difference of greater than or equal to 2 or less than or equal to 0.5 and a *p*-value of less than 0.05 across all cells cultured in native compared to standard conditions. The TCGA survival analysis was performed using the TCGA bioportal [47].

### Mitochondrial sequencing

Sequencing of mitochondrial DNA was performed using the REPLI-g Mitochondrial DNA Kit (Qiagen, Hilden, Germany) and the MiSeq System (Illumina, San Diego, CA, USA). Read alignments was performed against the Revised Cambridge Reference Sequence (rCRS) (gi|251831106|ref|NC_012920.1) [48] using BWA v0.7.15-r1140 [49]. This was followed by duplicate read marking by Picard tools v1.134 (http://broadinstitute.github.io/picard). Local insertion and deletion realignment was performed using GATK IndelRealigner v3.6 [50], followed by base recalibration using GATK BaseRecalibrator. Unpaired variant calling was performed using GATK HaplotypeCaller [50] with cohort Joint genotyping. Variant filtering was performed using GATK Variant Filtration according to standardized criteria. Variants were annotated with the Variant Effect Predictor (VEP) [51] perl script version 80 that references the Ensemble release 80 database.

### Statistical analysis

Two-tailed Student *t*-test and one- or two-way Analysis of Variance (ANOVA) or multiple comparisons were used where appropriate. The Kaplan–Meier method was used in survival analyses, and the log-rank test was used for survival comparison. Statistical significance was achieved when *p* < 0.05.

## Abbreviations

HCC: hepatocellular carcinoma
TCGA: The Cancer Genome Atlas
